# Molecular Dynamics Study on the Diffusion Behavior of Water Molecules and the Dielectric Constant of Vegetable/Mineral Oil Blends

**DOI:** 10.3390/molecules28031067

**Published:** 2023-01-20

**Authors:** Manqing Zhao, Bo Zhang, Jianfei Li, Qiankai Zhang, Huaqiang Li

**Affiliations:** 1 School of Electronics and Information, Xi’an Polytechnic University, Xi’an 710048, China; 2 State Key Laboratory of Electrical Insulation and Power Equipment, Xi’an Jiaotong University, Xi’an 710049, China

**Keywords:** mineral oil, vegetable oil, water content, dielectric constant, molecular simulation

## Abstract

Insulating oil plays a crucial role in internal insulation of oil-impregnated transformers. It has been demonstrated in a variety of experimental studies that mineral oil (MO) and vegetable oil (VO) can be blended in different ratios to improve insulation properties; however, the mechanisms underlying this phenomenon remain unclear. In this study, a molecular dynamics (MD) simulation approach was used to investigate diffusion of water molecules in VO/MO blends and dielectric constants of a mixture. The results show that the diffusion coefficient of water molecules is negatively correlated with the proportion of VO; thus, addition of VO helps to improve the insulation properties of a mixture. Due to introduction of strong polar functional groups, a decrease in the diffusion behavior of water molecules can be attributed to an increase in the interaction energy and formation of hydrogen bonds between water molecules and the mixed oil system. There is a direct correlation between the dielectric constant of a mixture and VO content; however, it is very sensitive to water content. The presence of strong polar water molecules or functional groups in a mixture leads to an increase in the dielectric constant, which results in a reduction in insulating properties. Accordingly, presence of polar groups plays an important role in determining the insulating properties of a mixture. To increase the insulation performance of a mixture, it is important to consider the diffusion-inhibiting and dielectric effects of the stronger polar groups in vegetable oil compared to those in mineral oil.

## 1. Introduction

An oil-impregnated power transformer is the core equipment for power conversion and transmission, and insulation performance directly affects safe and stable operation of the power system. In addition to providing insulation, transformer oil also plays a role in heat dissipation and cooling. However, in the presence of long-term electric fields and environmental factors, such as temperature and moisture, degradation of oil may result in transformer failure [1,2,3]. Therefore, maintaining the dielectric properties and insulation performance of insulating oil is vital for ensuring long-term transformer reliability. The oils used in transformers usually include vegetable oil (VO) and mineral oil (MO), with the latter currently being widely used in China. MO has excellent electrical and physicochemical properties; however, it also has some disadvantages, including low flash point, poor biodegradability, and non-regenerative properties [4,5]. The flash point of VO is higher than that of MO, and its lower carbon content makes it a more environmentally friendly alternative to MO [6,7,8]. As ultra-high-voltage (UHV) power transmission technology advances, the voltage level of transmission equipment increases, placing greater demands on insulating oils. Consequently, extensive research has been conducted to improve the insulating properties of transformer oil using methods such as nanoparticle doping, adding antioxidants, and blending VO and MO. Most commonly, insulating oil was doped with nanoparticles to improve the dielectric properties. Nagendran et al. [9], Du et al. [10], and Katiyar et al. [11] investigated the properties of insulating oil modified by nanoparticles and found that introduction of nanoparticles not only improved thermal conductivity but also increased breakdown voltage. Nanoparticles introduced moisture into oil, which adversely affects its insulation properties. Tian et al. [12,13] investigated the effects of nanoparticles on the diffusion behavior of water molecules in insulating oil based on molecular dynamics (MD) simulations. They found that nanoparticles can inhibit diffusion of water molecules and improve insulation properties by reducing their free volume fraction. In addition, Hao et al. [14] found that adding antioxidants to insulating oils can enhance their insulating properties by suppressing acid value, thereby delaying the aging process. Although addition of nanoparticles and antioxidants can improve the performance of insulating oil, introduction of impurities will adversely affect the interfaces between oil and other substances (e.g., paper and metal) and may induce partial discharge in severe cases. Therefore, to eliminate the influence of impurities on oil-paper insulation, some researchers have proposed blending VO and MO to improve insulation performance. Perrier et al. [15] found that adding 20 wt% synthetic ester to VO can improve its stability and water solubility without affecting its viscosity, while Lyutikov [16] and Karthik et al. [17] found that adding 10–50 wt% synthetic ester to MO can improve its AC breakdown voltage. It has been shown in numerous experimental studies that blending VO and MO improves the dielectric properties of insulating oils. In spite of this, the mechanism that underlies this phenomenon is rarely discussed, especially because it is difficult to explain at the molecular level. In addition, water content in insulating oil is one of the key factors affecting its insulation performance and is closely related to the operating state of the transformer.

This paper studied the diffusion behavior of water molecules and dielectric constant of blended oil changes in different MO/VO mixture ratio models at 1 wt%, 2 wt%, and 3 wt% water content using the MD simulation method. This study elucidated the molecular modification mechanisms of mixed oil and provided theoretical support for rational choice of the type and proportion of mixed oil.

## 2. Molecular Modeling and Simulation Details

All our MD simulations in this study were performed using COMPASS forcefield with a timestep of 1 ps in different processes, including molecular modeling, structural optimization, and dynamic simulation of water diffusion in mixed oils.

### 2.1. Molecular Modeling of Vegetable/Mineral Oil Blends

#### 2.1.1. Vegetable Oil and Mineral Oil

**Figure 1:**
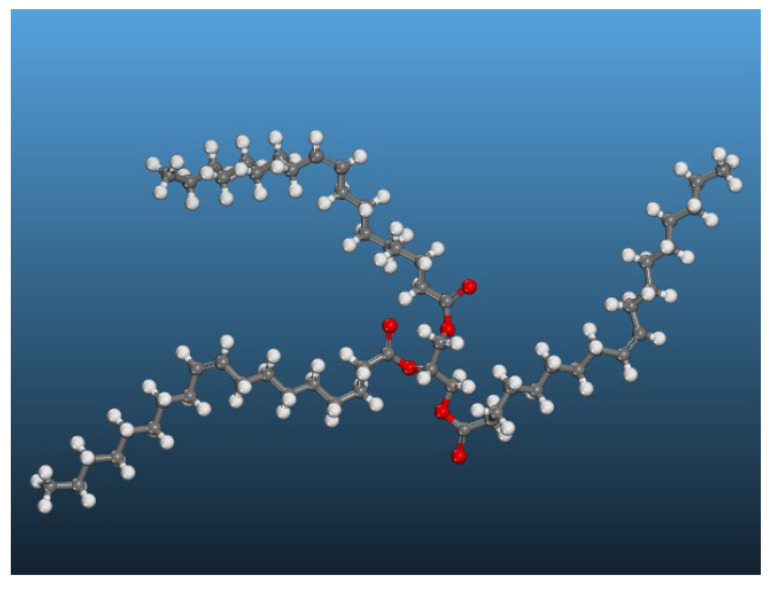
Atomic structure of triglyceride molecule.

The BIOTEMP insulating oil developed by ABB is one of the most commonly used VOs in oil-impregnated power transformers, containing more than 80 wt% of its component molecules in the form of triglyceride oleate (C_57_H_104_O_6_), along with glycerate (C_57_H_92_O_6_), trienoic acid triglycerides (C_57_H_92_O_6_), saturated triglycerides (C_39_H_74_O_6_), and antioxidants [18]. Thus, this type of oil was chosen to represent the VO counterpart in this study, using the oleic triglyceride molecule as its component [19], as shown in Figure 1, wherein the red, gray, and white balls represent oxygen, carbon, and hydrogen atoms, respectively.

The MO counterpart in this study was modeled based on Karamay 25^#^ insulating oil, with its primary constituents of saturated hydrocarbons, such as C_12_H_26_, C_14_H_28_, C_13_H_24_, C_16_H_28_, and C_16_H_26_, according to a previous report [20]. Therefore, typical paraffin and naphthenic hydrocarbon molecules were selected as representative component molecules of the MO, as shown in Table 1.

**Figure 2:**
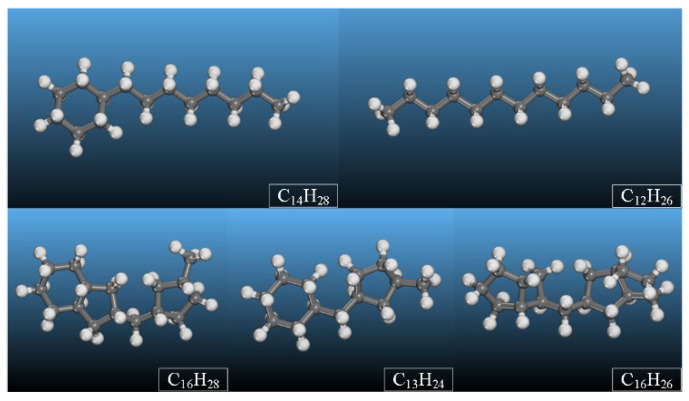
Atomic structures of the main constituent molecules in the Karamay 25^#^ insulating oil.

Figure 2 shows the atomic structures of the component molecules in the Karamay 25^#^ insulating oil listed in Table 1.

#### 2.1.2. Molecular Modelling Details

**Figure 3:**
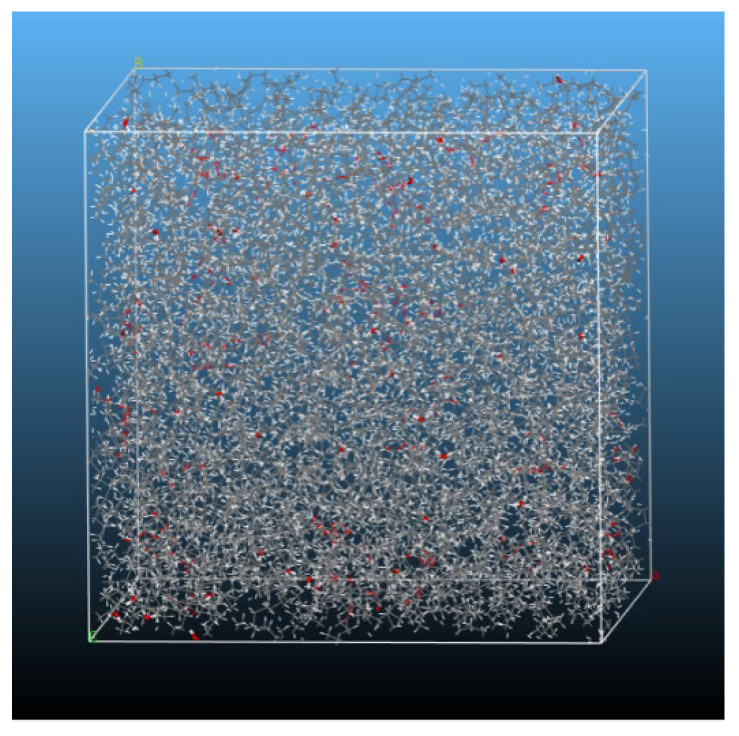
30 wt% VO mixed oil model with 1wt% of water content.

In order to investigate the effects of blending ratios of VO and MO on water diffusion behavior and the mixture’s dielectric constant, mixed oil models containing a water content of 1 wt%, 2 wt%, or 3 wt% were constructed using the Amorphous Cell module in MS. A total of 18 nanoscale molecular models of mixed oil were analyzed with MO:VO mass ratios ranging from 0:10, 1:9, 2:8, 3:7, 4:6, and 5:5. These models are identified according to their relative mass fractions of VO components, which are 0 wt% VO, 10 wt% VO, 20 wt% VO, 30 wt% VO, 40 wt% VO, and 50 wt% VO, respectively. The water model we used in our simulations was a simple three-point model, which was embedded in COMPASS forcefield [21,22,23], resulting in a model size of approximately 59 Å × 59 Å × 59 Å. As an example, Figure 3 illustrates the initial structure of the 30 wt% VO mixed oil model with water content of 1 wt%.

### 2.2. Structural Optimization and Dynamic Simulation Details

In the Amorphous Cell module, the initial structure of a polymer is constructed using the Monte Carlo (MC) algorithm, which results in relatively high energy. Therefore, further optimization and relaxation are required to reduce the overall energy of the model in order to achieve equilibrium. The COMPASS forcefield was used for the structural optimization, relaxation, and dynamic simulation processes, and the exact potential forms and part of the parameterizations for COMPASS forcefield were reported in Supplementary Materials [24,25,26,27,28]. A time step of 1 fs was set, and the periodic boundary conditions were applied to all directions. The COMPASS forcefield was used for the structural optimization, relaxation, and dynamic simulation processes, and the exact potential forms and part of the parameterizations for COMPASS forcefield were reported in Supplementary Materials. A time step of 1 fs was set, and the periodic boundary conditions were applied to all directions. The electrostatic force is calculated using the integral method of Ewald, and the van der Waals interaction we used in this study was specified by the 6–9 potential, with its exact forms stated in Supplementary Materials for COMPASS forcefield. The structural optimization process includes geometric optimization and annealing optimization. The Smart method with a target energy convergence value of 10^−4^ kcal/mol and a maximum number of iterations of 10,000 was used for the geometry optimization of our initial mixed oil models. Afterward, the optimized structure was subjected to five cycles of annealing between 343 K and 500 K in order to overcome the restrictions introduced by the dihedral potential barrier of the molecular chains. The oil temperature in an oil-impregnated transformer is normally maintained at around 70 °C during operation, so an initial temperature of 343 K was selected for our simulation.

The annealed optimized molecular configuration was then subjected to dynamic relaxation process in the canonical (NVT) ensemble and isothermal–isobaric (NPT) ensembles in succession. For the NVT simulation, the optimized models were relaxed for 50 ps, holding the system temperature at 343 K using Nosé–Hoover thermostat. For the NPT simulation, the Nosé–Hoover thermostat and the Berendsen barostat were selected, and another dynamic relaxation of 50 ps was performed over all configurations at T = 343 K, *p* = 1 atm. A step size of one fs was used throughout the relaxation process. The energy plots were all conserved during system optimization and relaxation processes as mentioned above (see Supplementary Materials for more detailed information).

The mixed oil models reached equilibrium after optimization and relaxation (see Figure S1 in Supplementary Materials), and the resulting average densities were around 0.7 g/cm^3^, which is consistent with experimental results. Finally, the optimized models were subjected to NVT simulations using the Nosé–Hoover thermostat for 500 ps at 343 K to analyze the water diffusion behavior as well as the dielectric constants of mixed oils.

## 3. Results and Discussion

### 3.1. Diffusion Behavior of Water Molecules

Oil-paper insulation in oil-impregnated transformers gradually deteriorates over time under the influence of various environmental factors, such as temperature, humidity, and electrical fields, resulting in reduced insulation performance and occurrence of insulation faults. Among the factors mentioned above, the water content in insulating oil is generally regarded as the greatest threat to its insulation properties. Therefore, the effect of mixed oil composition on diffusion behavior of water molecules was investigated, and the physical mechanisms behind it were explained in terms of interaction energy, hydrogen bonding, free volume fractions, as well as centroid trajectories of water molecules.

#### 3.1.1. Diffusion Coefficient of Water Molecules

Particle diffusion behavior can be described by the mean square displacement (*MSD*) curve, which represents the average distance between the positions of all particles at time *t* and their initial positions [29]. The *MSD* is measured over time to determine diffusing ability of the target particle(s) in the specific system, which can be expressed as below:

(1) $$MSD=\langle {\left|\vec{r_{i}}(t)-\vec{r_{i}}(0)\right|}^{2}\rangle$$

where $\vec{r_{i}}\left(t\right)$ and $\vec{r_{i}}\left(0\right)$ are the position vectors of the particle at *t* = t and *t* = 0, respectively, and < > represents the statistical average of the movement trajectories.

**Figure 4:**
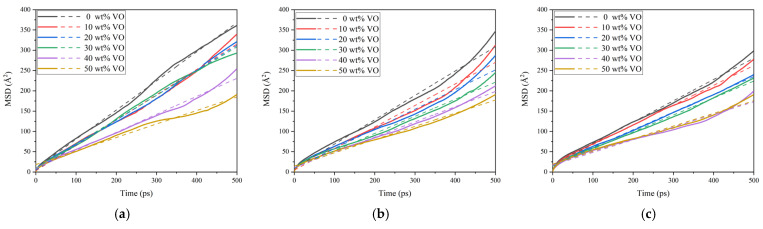
*MSD* curves of water molecules in each mixed oil model with different water contents: (**a**) 1 wt%; (**b**) 2 wt%; (**c**) 3 wt %.

The diffusion coefficient is an important parameter for characterizing the diffusivity of particles. For example, a higher diffusion coefficient of water molecules in oil indicates larger directional or non-directional displacement of water molecules. The greater diffusion ability of water molecules makes it easier for them to overcome the potential barriers imposed by their surroundings, facilitating motion and aggregation of water molecules under external electric fields, which would ultimately deteriorate the electrical and insulating performance of transformer oil. The diffusion coefficient *D* of water molecules in oil can be calculated as below [30]

(2) $$D=\frac{1}{6N}\underset{t\to \infty }{\lim}\frac{d}{dt}\sum_{i=1}^{N}{\left|{\vec{r}}_{i}(t)-{\vec{r}}_{i}(0)\right|}^{2}=\frac{a}{6}$$

where *a* is the slope of the fitting line for the *MSD* curve. Figure 4 shows the *MSD* results for the mixed oil models with 1 wt% to 3 wt% water content at 343 K, respectively. The solid lines represent the *MSD* curves of the water molecules in the mixed oil models with different VO ratios, and the dashed lines are the linear fitting lines of the corresponding *MSD* curves.

In Table 2, Table 3 and Table 4, the slope of the fitting line for the *MSD* curve (*a*) and the diffusion coefficient of water molecules (*D*) are presented corresponding to the data in Figure 4. These results show that, for a given water content, the diffusion coefficient of water molecules gradually decreases as the proportion of VO increases. When the water content is 1 wt%, the value of *D* in the mixed oil gradually decreases from 0.1196 Å^2^/ps in pure MO (0 wt% VO) to 0.0577 Å^2^/ps in the 50 wt% VO mixture, while, at 3 wt% water content, the corresponding values are 0.0901 Å^2^/ps and 0.0529 Å^2^/ps, respectively. The reason is that the constituent molecules of VO are triglycerides of oleic acid, which contains several stronger atom groups, such as carbonyls (C=O) and ethers (R-O-R), compared to the hydrocarbon atom groups in mineral oil, as illustrated in Figure 1. The presence of carbonyl and ether groups in VO counterparts could both promote formation of hydrogen bonds and increase the interaction energy in the mixed oil, thereby limiting diffusion of water molecules. For a given water content, a higher proportion of VO in the mixed oil model results in a greater number of the stronger polar groups from VO introduced into the system, which reduces the diffusion coefficient of water molecules.

For mixed oils with a given proportion of VO, the diffusion coefficient of water molecules is negatively correlated with the water concentration in oil. Strong polarity of water molecules promotes formation of hydrogen bonds and increases interaction energy in the system, which inhibits thermal motion and diffusivity of water molecules. Although increasing the water concentration in transformer oil decreases the diffusion coefficient of water molecules, the effect of increasing the water concentration on the insulation performance of transformer oil outweighs the effect of decreasing the diffusion capacity. As an example, for the 30 wt% VO mixed oil, increasing the water content from 1 to 3 wt% (i.e., by a factor 3) will result in a decrease of 30 wt% in the diffusion coefficient of water molecules, from 0.1009 Å^2^/ps to 0.0711 Å^2^/ps. Therefore, the overall insulation performance still shows a downward trend with increasing water content. The findings above coincide with the diffusion coefficient of water molecules in vegetable oil reported in [19], which was smaller than that in mineral oil. In addition, introduction of polar groups to which the water molecule would interact would slow the dynamics behavior of water molecules, as reported in [31], which is also in consistency with our statements above.

Further quantitative analysis of the impact of water and VO content on the diffusion behavior of water molecules in the mixed oil was carried out by calculating the number of hydrogen bonds and the interaction energy between the water and oil molecules.

#### 3.1.2. Hydrogen Bonds

In contrast to chemical bonds, hydrogen bonds refer to non-bonding interactions between hydrogen atoms and strong electronegative atoms or atom groups, whose strength lies between covalent bonds and van der Waals interactions [32]. The number of hydrogen bonds formed in a system is closely related to the diffusivity of the investigated particles. In general, the more hydrogen bonds formed in a system, the stronger the attractive interactions between molecules, thereby limiting their diffusion.

**Figure 5:**
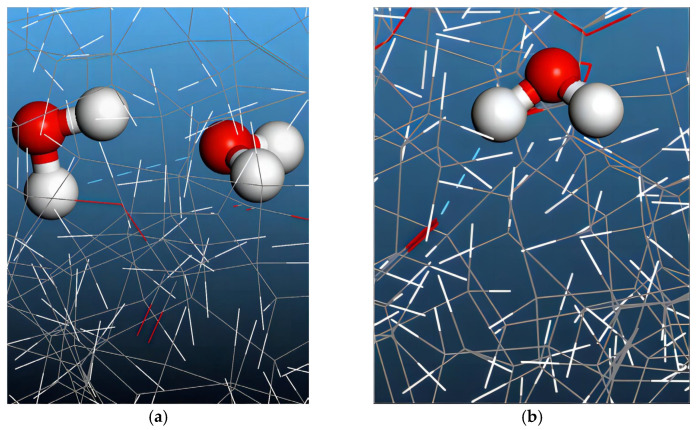
H-bonds formed in mixed oil model: (**a**) hydrogen bonds formed between water molecules; (**b**) hydrogen bonds formed between VO molecules and water molecules.

According to the structural characteristics of the constituent molecules in mixed oil, two types of hydrogen bonds could be formed: In the first type, the hydrogen bonds formed between water molecules, as shown in Figure 5a. The second type of hydrogen bond occurs between the oxygen atoms from the stronger polar groups in VO molecules and hydrogen atoms in the water molecules, as shown in Figure 5b.

**Figure 6:**
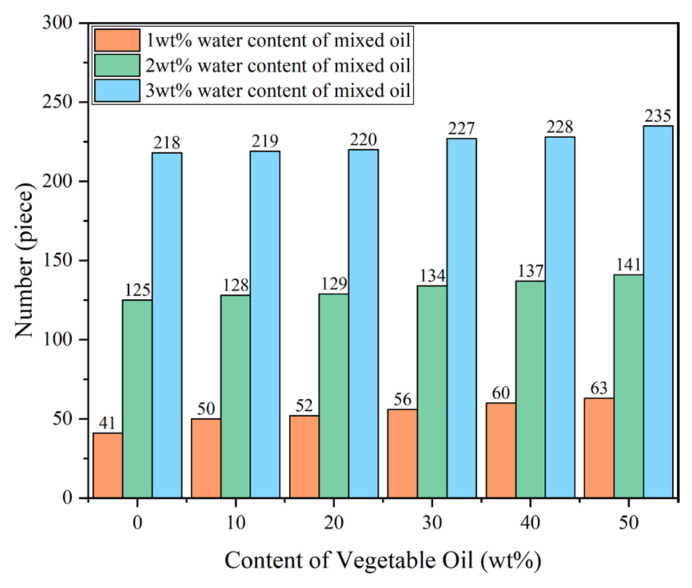
The number of H-bonds in each mixed oil model.

To qualify as a hydrogen bond, the hydrogen bond donor atom (H atom in our case) and acceptor atom (O atom in this case) should be separated by 2.5 Å or less and the do-nor–acceptor–acceptor antecedent angle should be larger than 100°, which is also a commonly used method in determining the hydrogen bonding in a liquid system, as reported in [33]. The number of hydrogen bonds in each frame of the 500 ps dynamic trajectory of the different mixed oil models was calculated and averaged using a script written in Perl, with the results shown in Figure 6.

The results show that, for both levels of water content, the number of hydrogen bonds in mixed oil models increases as the proportion of VO increases. For 1 wt% water content models, the number of hydrogen bonds increased from 41 in 0 wt% VO to 63 in 50 wt% VO, and, when the water content is 2 wt%, the number of hydrogen bonds increased from 125 in 0 wt% VO to 141 in 50 wt% VO, whereas, for 3 wt% water content models, the corresponding numbers were 218 and 235, respectively. Regardless of water content, along with an increase in VO proportion, the number of stronger polar groups increased in the mixed oil, leading to formation of more hydrogen bonds. Due to hydrogen bonding, water molecules are subjected to an increased level of attractive interactions with oil molecules, resulting in a reduction in their diffusion coefficient in the system. This is consistent with the calculation results in Figure 4. It should be noted, however, that the number of hydrogen bonds is significantly higher in the mixed oil with 3 wt% water content compared to those with 1 wt% and 2 wt% water content. Since the water molecule has a much smaller molecular weight than the oleic triglyceride molecule in VO, increasing water content introduces a significantly higher number of polar molecules compared to increasing VO proportion in mixed oil.

Furthermore, in order to investigate the contributions of the two different types of hydrogen bonds on the total number hydrogen bonds in mixed oil, the average number of hydrogen bonds formed between water molecules and between water and vegetable oil molecules in the system were calculated. The results are shown in the Table 5, Table 6 and Table 7. HB_W-W_ refers to the hydrogen bonds formed between water molecules, as illustrated in Figure 5a, and HB_W-V_ refers to the hydrogen bonds formed between water molecules and vegetable oil molecules, as illustrated in Figure 5b. For a given VO proportion in mixed oil, both types of hydrogen bonds increased with increasing water content, indicating that movement of water molecules is increasingly constrained in terms of hydrogen bonding with the increase in water content. Additionally, for a given water content in mixed oil, the number of hydrogen bonds between water molecules and vegetable oil molecules showed an increasing trend with increasing VO proportion in mixed oil, which indicates that formation of hydrogen bonds between water molecules and vegetable oil molecules accounts for an increase in total hydrogen bonds in mixed oil with increasing VO proportion, as shown in Figure 6. The findings above explained our diffusion coefficients results in different mixed oil models in Table 2, Table 3 and Table 4 in terms of hydrogen bonding.

To further analyze the influence of hydrogen bonds on the diffusion coefficient of water molecules, the number of hydrogen bonds formed per unit water molecule was calculated, as shown in Table 8, Table 9 and Table 10. The increased proportion of VO did increase the number of hydrogen bonds in the mixed oil to some extent. In addition, the number of hydrogen bonds per unit water molecule in mixed oil models also increased with the increase in water content. As a result, increasing the water content could limit not only the overall thermal movements of all molecules in the system but could also reduce the thermal movements of the water molecules in transformer oil. This result explains the decrease in diffusion coefficient of water molecules with increasing water content and VO content in the system from the perspective of hydrogen bond formation.

#### 3.1.3. Interaction Energy

In the oil–water system, the interaction energies between water and oil molecules contribute significantly to the diffusivity of water molecules in terms of intermolecular interactions. When quantifying these interactions, a positive or negative sign indicates either repulsive or attractive forces, and the magnitude indicates the interaction strength. In an oil–water system, the total energy includes both kinetic and potential energy of all molecules in the system. The potential energy can be categorized into electrostatic potential energy and van der Waals potential energy based on the different types of interactions. Moreover, it can also be classified as interaction energy between particles of the same type as well as particles of different types. Therefore, the interaction energy between oil and water molecules can be expressed as follows:

(3) $$E_{int}=E_{T}-(E_{O}+E_{W})$$

where *E_int_* is the interaction energy between the oil molecules and water molecules, *E_T_* is the total potential energy of the whole oil–water system, *E_O_* is the potential energy of the mixed oil, including the interaction energy among six types of constituent molecules in mixed oil models, as shown in Figure 1 and Figure 2, while *E_W_* is the potential energy between the water molecules themselves.

A separate account of the interaction energy between MO or VO molecules and water molecules was made by dividing the oil–water model into “model 1”, composed of VO and water molecules, and “model 2”, composed of MO and water molecules, in order to calculate the interaction energy between the mixed oil and water molecules in terms of the different MO or VO contributions. Then, Equation (3) can be rewritten as follows:

(4) $$E_{\mathrm{int}-T}=E_{\mathrm{int}-M}+E_{\mathrm{int}-V}$$

where *E_int-T_* represents the total interaction energy between the mixed oil and water molecules, *E_int-M_* represents the interaction energy between MO and water molecules, and *E_int-V_* represents the interaction energy between VO and water molecules. Through Perl scripting, the potential energies of *E_T_*, *E_O_*, and *E_W_* in each frame of the dynamic trajectory were calculated separately for “model 1” and “model 2” and then averaged. The resulting values of *E_int-V_*, *E_int-M_*, and *E_int-T_* are shown in Table 11, Table 12 and Table 13.

For a given water content, the interaction energy between water molecules and mixed oil molecules is negative, which indicates an attractive interaction. In addition, the interaction energy decreases (i.e., becomes more negative) as the proportion of VO increases, indicating that the attraction between water and oil molecules increases with the increase in VO content in mixed oil. Thus, the movement of water molecules becomes more restricted with increasing VO in mixed oil, resulting in a reduction in their diffusion coefficient, which confirms the results of the diffusion coefficient calculations shown in Figure 4. Meanwhile, the calculation results for the interaction energy of water molecules with VO and MO in Table 11, Table 12 and Table 13 show that the changing rate of *E_int-V_* is significantly higher than that of *E_int-M_*. This is due to the stronger interactions between polar groups (carbonyls and ethers) in VO and water molecules than between MO and water molecules.

In order to quantitively investigate the influence of VO contents in mixed oil on diffusion behavior of an individual water molecule, the average interaction energy between a single water molecule and a single VO molecule was calculated and shown in Table 14. For a given water content, it can be inferred from the calculated interaction energy between a single water molecule and a single VO molecule that with the increase of VO content in the mixed oil, the attraction ability of VO molecules to water molecules will increase, thus limiting the mobility of water molecules. A similar phenomenon could be found for the results of interaction energy between a single molecule and VO molecule in mixed oil for a given water content. An increase in water content in mixed oil also contributes to enhancement of the attraction between a single water molecule and the VO component molecule, which also restricts the mobility of water molecules in mixed oil in light of the VO constituent in mixed oil. The calculations in Table 14 directly correspond to the diffusion coefficient of water molecules in mixed oil in Table 2, Table 3 and Table 4, illustrating the significant impact of VO constituent on the diffusion behavior of water molecules in mixed oil.

Due to the strong polarity of water molecules, an increase in their proportion in mixed oil will increase the overall interaction energy of the entire system, including the contributions of hydrogen bonds. The statistical calculations of hydrogen bonds in Table 8, Table 9 and Table 10 indicate that the average number of hydrogen bonds per water molecule increases with increasing water content, which intensifies the attraction between oil molecules and water molecules, resulting in reduced diffusion coefficients of water molecules.

#### 3.1.4. Fractional Free Volume (FFV)

In order to further analyze the decrease in the diffusion coefficient of water molecules in the mixed oil, the FFV of water molecules in the mixed oil under different VO proportions was calculated. According to Fox and Flory’s free volume theory [34], the total volume ($V_{t}$) of insulating materials can be divided into occupied volume ($V_{o}$) and free volume ($V_{f}$). The FFV formula is shown in Equation (5):

(5) $$FFV=\frac{V_{f}}{V_{f}+V_{0}}\times 100\%$$

The larger the FFV of the system, the more space available for particle displacement, which is conducive to diffusion and displacement of particles in the system due to thermal motion. At 1 wt%, 2 wt%, and 3 wt% water content, the fractional free volume calculation results of the models with different mixing proportions are shown in Figures S3–S5 (considering the paper length, the free volume diagram is provided in Supplementary Materials); the blue area represents the free volume of water molecules, and the gray area represents the occupied volume of the interface structure. Compared to the models with 1 wt% water content, the free volume of water molecules decreased in all models with 3 wt% water content. It is worth noting that, with the increase in VO content under the same water content, the free volume of water molecules also showed a trend of gradual decrease owing to the large number of strong polar groups introduced by water and vegetable oil molecules. These polar groups may combine oil molecules and water molecules more, resulting in a decrease in the free volume of water molecules in oil. The calculated law of free volume fraction is consistent with the law of diffusion coefficient, which explains one of the causes of decreasing water molecular diffusion behavior with increased water content and VO content. To reflect the difference of free volume in different models more intuitively, the free volume fraction of water molecules in each model at moisture contents of 1 wt%, 2 wt%, and 3 wt% were calculated, respectively. The results are shown in Table 15, Table 16 and Table 17.

The results show that the free volume of water molecules gradually decreases and the occupied volume increases with the increase in the mass fraction of VO. At the moisture content of 1 wt%, 2 wt%, and 3 wt%, the FFV decreased from 12.4%,12.2%, and 12.1% (0 wt% VO) to 8.5%, 8.4%, and 8.1% (50 wt% VO), respectively. This verifies the conclusions of this section.

#### 3.1.5. Centroid Trajectories of Water Molecules in Mixed Oil

To describe the effect of the percentage of VO more intuitively on the diffusion behavior of water molecules, the centroid trajectories of water molecules at 500 ps in the NVT process were calculated using a Perl script. Figures S6–S8 (considering the paper length, the free volume diagram is provided in Supplementary Materials) illustrate the centroid trajectories of water molecules in the mixed oil at 1 wt%, 2 wt%, and 3 wt% water content. These results show that, at a fixed water content, the movement range of the water molecules gradually decreases as the proportion of VO increases; taking 10 wt% VO content as an example, when the water content increases from 0 wt% to 50 wt%, the motion range of water molecules on the X and Z axes decreases from 4 Å to about 3 Å, and the motion range on the Y axis decreases from 5 Å to 3 Å. This demonstrates a rise in VO content limiting their diffusivity, which is consistent with the earlier results. Furthermore, at a fixed proportion of VO, the movement range of the centroid trajectory of water molecules in the mixed oil is smaller at the relatively higher water content (3 wt%) system; taking 1 wt% water content as an example, when the vegetable oil content increases from 0 wt% to 50 wt%, the motion range of water molecules on the X and Z axes decreases from 4 Å to about 3 Å, and the motion range on the Y axis decreases from 5 Å to 3 Å, which also confirms the previous results.

The diffusion behavior of water molecules in mixed oil shows that, as the proportion of VO increases, the diffusion of water molecules is inhibited, which reduces the moisture in the oil, thereby improving the insulating properties of the mixed oil. This phenomenon is closely related to introduction of stronger polar groups on the oleic triglyceride molecules in VO into the mixture. As the proportion of VO increases, the polar groups promote formation of hydrogen bonds with water molecules and increase the interaction energy, thus limiting the diffusion of water molecules in the oil. Therefore, when considering enhancement of insulation performance through blending VO and MO, the type and proportion of VO added can be selected according to the type and number of polar groups in the VO.

### 3.2. Static Dielectric Constant of Mixed Oil

The dielectric constant *ε* is an important parameter reflecting the polarizing and insulating properties of dielectric materials. It includes the static dielectric constant *ε*_s_ as well as the optical dielectric constant *ε*_∞_. *ε*_s_ reflects the relative dielectric constant and the relaxation polarization of a material at a constant electric field, which is closely related to the molecular structure of the material; *ε*_∞_ represents the relative dielectric constant at the frequency of the applied electric field equal to the optical frequency, which is related to the instantaneous displacement polarization of the material. In order to study the effect of the molecular structure and water content of the mixed oil on its dielectric properties, the value of *ε*_s_ was calculated. The fluctuation method and the applied external field method are two commonly used MD methods for calculating *ε*_s_ [35]. The former method relates *ε*_s_ with the rise and fall of the electric dipole moment within the material, which is solved by applying an infinitesimal electric field excitation to the simulated system. In response to such fluctuations in the electric dipole moment within the system, statistical averaging is used to resolve the issue. As the external field excitation used in the method is infinitesimal, the solution can also be obtained without applying an external electric field. The applied external field method, on the other hand, induces polarization within the system by applying an enhanced external electric field and is suitable for materials with zero permanent dipole moment [36,37]. In the present study, since a non-zero permanent dipole moment exists in the mixed oil system, *ε*_s_ was calculated using the fluctuation method. Accordingly, the fluctuation of the electric dipole moment of the constituent molecules in the system can be expressed as follows:

(6) $$\begin{array}{l} \langle {\left|M\right|}^{2}\rangle -{\left|\langle M\rangle \right|}^{2}=\langle {\left|M_{x}\right|}^{2}\rangle -{\left|\langle M_{x}\rangle \right|}^{2}+\langle {\left|M_{y}\right|}^{2}\rangle \\ -{\left|\langle M_{y}\rangle \right|}^{2}+\langle {\left|M_{z}\right|}^{2}\rangle -{\left|\langle M_{z}\rangle \right|}^{2} \end{array}$$

where *M* represents the total electric dipole moment of the system, *M*_x_, *M*_y_, and *M*_z_ are its components in the *x*, *y*, and *z* axes, and < > represent the statistical averaging in the dynamical trajectory.

According to the literature [38], the relationship between the electric dipole moment fluctuation and the static dielectric constant is provided by [39,40,41]:

(7) $$\frac{\langle {\left|M\right|}^{2}\rangle -{\langle \left|M\right|\rangle }^{2}}{3\varepsilon_{0}Vk_{B}T}=\frac{\left(2\varepsilon_{RF}+1\right)\left(\varepsilon -1\right)}{\left(2\varepsilon_{RF}+\varepsilon \right)}$$

where *M* represents the total electric dipole moment of the system, *ε*_0_ is vacuum permittivity, *V* is the volume of the system, *k*_B_ is the Boltzmann constant, *T* is the simulated temperature, *ε* is the dielectric constant of the system (specifically, the static dielectric constant *ε*_s_), and *ε*_*RF*_ is the dielectric continuum spectrum within the system. In the fluctuation method, *ε*_*RF*_→∞ hence, *ε*_s_ can be expressed as follows:

(8) $$\varepsilon_{s}=1+\frac{\langle {\left|M\right|}^{2}\rangle -{\langle \left|M\right|\rangle }^{2}}{3Vk_{B}T\varepsilon_{0}}$$

**Figure 7:**
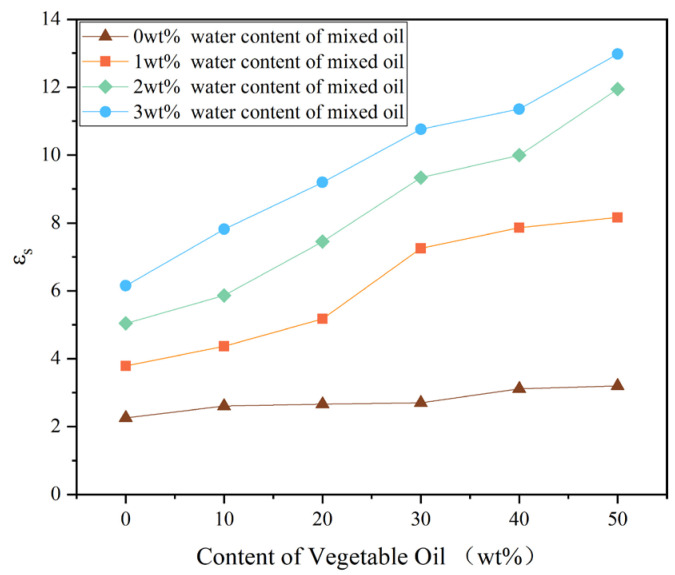
Dielectric constant of mixed oil models.

Using Equations (6) and (8), the *ε*_s_ of the mixed oil with different VO fractions at 0 wt%, 1 wt%, 2 wt%, and 3 wt% water content were calculated by Perl scripting, with the results shown in Figure 7. In contrast to calculation of parameters such as diffusion coefficients and interaction energies, prediction of the dielectric properties of a material typically requires longer simulations due to the large molecular sizes of the model and the low degree of molecular orientation fluctuations. In such cases, a reasonable simulation duration should be used based on the convergence time. Based on a mixed oil model with 50 wt% VO content at 1 wt% moisture content, a molecular dynamic simulation test was conducted for 500 ps. It was found that the frame-by-frame output of the dielectric constant data fluctuated severely during the initial 300 ps and gradually stabilized afterwards. Consequently, a simulation time of 500 ps is sufficient to ensure convergence and accuracy of the static dielectric constant of our mixed oil models. As shown in Figure 7, as the proportion of vegetable oil is increased, the calculated dielectric constant of the mixed oil increases from 2.250 (0 wt% VO) to 3.199 (50 wt% VO). In this simulation, it is demonstrated that mixed oil without moisture content has a static dielectric constant between mineral oil and vegetable oil, which is consistent with the expectation (mineral oil’s dielectric constant is 2.2, while vegetable oil’s is 3.2). In addition, the static dielectric constants of the calculated pure mineral oil (0 wt% VO) at a water content of 0 wt%, 1 wt%, 2 wt%, and 3 wt% are 2.250, 3.784, 5.040, and 6.152, respectively. The static dielectric constant of pure mineral oil under different water content was calculated. As expected, the calculated value is higher than the measured value of the power frequency dielectric constant under moist free condition, which is mainly attributed to the influence of water content and low voltage frequency. Especially, a comparison of the effect of voltage frequency and impurities of water on dielectric constant shows that impurities have a greater influence. With increasing water content in mineral oil, the number of particles participating in polarization per unit volume increases. Water is a strongly polar molecule with a relative dielectric constant of 81; thus, a higher concentration increases its dielectric constant. In addition, the static dielectric constant calculated in this paper is in accordance with the numerical trend in the low frequency range (<10^−1^ Hz) measured by other scholars; that is, with the increase in water content in mineral oil, the values of both the calculated static dielectric constant and the measured dielectric constant in the low frequency range (<10^−1^ Hz) increased significantly, with a similar growth law [42,43]. It has been reported that, as the water content of MO increases, the dielectric constant increases more drastically at low frequencies (<10^−1^ Hz) than at high frequencies. For a given proportion of VO in mixed oil models, the calculated *ε*_s_ values were also found to be positively correlated with the water content, which is consistent with the results for pure MO, further indicating that the water content in the oil significantly influences the dielectric constant at low frequency.

When the water content is the same, the calculated value of the static dielectric constant *ε*_s_ of the mixed oil increases significantly with the increase in the content of VO. Compared with MO, the molecules of VO contain more stronger polar groups (carbonyl and ether groups) and thus more particles participating in the polarization process, resulting in an increase in the dielectric constant of the mixed oil with an increase in the proportion of VO. Meanwhile, ε_s_ of mixed oils with different water contents increases significantly with an increase in the proportion of VO, indicating that water content has a significant effect on the dielectric constant of VO at low frequency, which is more obvious than that of MO, which could be attributed to the following two factors: with an increase in water content, the number of particles participating in polarization per unit volume increases, leading to an increase in the dielectric constant of the system. In addition, the strong interaction between the stronger polar groups in VO and the water molecules further affects the polarization process of the molecules in the mixed oil, resulting in the dielectric constant of the oil at low frequency increasing significantly with the increase in water content. The effect of water content on the dielectric constant of VO in the low frequency domain has few reports, so the calculation conclusion should be further verified by experiments.

According to the calculation and analysis of the static dielectric constant above, the static dielectric constant of the mixed oil gradually increases with an increase in the proportion of VO, and it is very sensitive to the moisture content, which has a negative impact on the dielectric properties of the mixed oil, which is closely related to introduction of the stronger polar groups (carbonyl and ether groups) in VO. With the increase in the proportion of VO, the increase in the number of stronger polar groups from VO leads to an increase in the static dielectric constant of the mixed oil.

## 4. Conclusions

In this study, the diffusion behavior of water molecules in different MO/VO blends and the dielectric constant of mixed oil were investigated using molecular dynamics simulations. These results help to explain the physical mechanism behind insulation properties of blends.

(1) As the proportion of VO in mixed oil increases, the diffusion coefficient of water molecules in the oil decreases. The presence of stronger polar groups in VO increases the number of hydrogen bonds and interaction energy between water molecules and mixed oil, which restricts diffusion of water molecules in the system. Therefore, increasing the proportion of VO can improve the insulation performance of mixed oil.
(2) The static dielectric constant increases significantly as the proportion of VO in mixed oil increases. Introduction of stronger polar groups in VO molecules increases the number of polarized particles per unit volume, which results in a significant increase in the static permittivity of the mixed oil. Therefore, increasing the proportion of VO has a negative effect on the insulation performance of mixed oil.
(3) The diffusion coefficient and static dielectric constant calculations reveal that introduction of the stronger polar groups present in VO plays a key role in determining the dielectric properties of mixed oil. Therefore, it is necessary to consider the type and number of polar groups of the added VO molecules when blending VO and MO for insulation modification. To improve the insulating properties of the mixed oil, it is necessary to consider the inhibitory effect of the polar groups on the diffusion behavior of water molecules and the positive effect on the dielectric constant.

This study provides an effective simulation method for studying the physical mechanisms of insulation modification in mixed oil and provides theoretical support for determining the most suitable oil types and composition of VO/MO blends.

## Figures and Tables

**Table 1:** Main components and their mass fractions of Karamay 25^#^ insulating oil.

Composition	Paraffin		Cyclic Hydrocarbons			In Total
		Single	Double	Tricyclic	Tetranuclear	
Mass Fractions	11.6	15.5	28.5	23.3	9.7	88.6

**Table 2:** The slope of the fitting line of *MSD* curve and the diffusion coefficient of water molecules in each mixed oil model with 1 wt% of water content.

VO Proportion	0 wt%	10 wt%	20 wt%	30 wt%	40 wt%	50 wt%
*a*	0.7176	0.6239	0.6141	0.5974	0.4440	0.3332
*D* (Å^2^/ps)	0.1196	0.1040	0.1024	0.0996	0.0740	0.0555

**Table 3:** The slope of fitting line of the *MSD* curve and the diffusion coefficient of water molecules in each mixed oil model with 2 wt% of water content.

VO Proportion	0 wt%	10 wt%	20 wt%	30 wt%	40 wt%	50 wt%
*a*	0.6004	0.5303	0.4843	0.4212	0.3746	0.3252
*D* (Å^2^/ps)	0.1001	0.0884	0.0807	0.0702	0.0624	0.0542

**Table 4:** The slope of fitting line of the *MSD* curve and the diffusion coefficient of water molecules in each mixed oil model with 3 wt% of water content.

VO Proportion	0 wt%	10 wt%	20 wt%	30 wt%	40 wt%	50 wt%
*a*	0.5245	0.4876	0.4367	0.4185	0.3129	0.3124
*D* (Å^2^/ps)	0.0874	0.0813	0.0727	0.0698	0.0522	0.0520

**Table 5:** The average number of different types of hydrogen bonds at 1 wt% water content.

VO Proportion	0 wt%	10 wt%	20 wt%	30 wt%	40 wt%	50 wt%
*HB_W-W_*	41	47	45	44	43	41
*HB_W-V_*	0	3	7	12	17	22

**Table 6:** The average number of different types of hydrogen bonds at 2 wt% water content.

VO Proportion	0 wt%	10 wt%	20 wt%	30 wt%	40 wt%	50 wt%
*HB_W-W_*	125	118	115	110	109	107
*HB_W-V_*	0	10	14	24	28	34

**Table 7:** The average number of different types of hydrogen bonds at 3 wt% water content.

VO Proportion	0 wt%	10 wt%	20 wt%	30 wt%	40 wt%	50 wt%
*HB_W-W_*	218	208	203	198	188	185
*HB_W-V_*	0	11	17	29	40	50

**Table 8:** The average number of H-bonds per water molecule in mixed oil models with 1wt% of water content.

VO Proportion	0 wt%	10 wt%	20 wt%	30 wt%	40 wt%	50 wt%
Averaged Number of H-bonds	0.683	0.819	0.852	0.918	0.984	1.033

**Table 9:** The average number of H-bonds per water molecule in mixed oil models with 2wt% of water content.

VO Proportion	0 wt%	10 wt%	20 wt%	30 wt%	40 wt%	50 wt%
Averaged Number of H-bonds	1.025	1.049	1.057	1.098	1.123	1.156

**Table 10:** The average number of H-bonds per water molecule in mixed oil models with 3wt% of water content.

VO Proportion	0 wt%	10 wt%	20 wt%	30 wt%	40 wt%	50 wt%
Averaged Number of H-bonds	1.172	1.198	1.203	1.247	1.253	1.291

**Table 11:** Interaction energy between mixed oil and water molecules with 1 wt% of water content.

VO Proportion	0 wt%	10 wt%	20 wt%	30 wt%	40 wt%	50 wt%
*E_int-M_* (kcal/mol)	−97.3	−84.3	−76.3	−54.2	−49.9	−46.6
*E_int-V_* (kcal/mol)	--	−53.7	−54.5	−127.0	−151.1	−157.6
*E_int-T_* (kcal/mol)	−97.3	−130.7	−138.0	−181.3	−201.0	−204.1

**Table 12:** Interaction energy between mixed oil and water molecules with 2 wt% of water content.

VO Proportion	0 wt%	10 wt%	20 wt%	30 wt%	40 wt%	50 wt%
*E_int-M_* (kcal/mol)	−181.9	−160.2	−143.7	−115.4	−105.9	−89.8
*E_int-V_* (kcal/mol)	--	−59.2	−107.8	−180.3	−196.7	−234.9
*E_int-T_* (kcal/mol)	−181.9	−219.4	−251.5	−295.7	−302.6	−324.7

**Table 13:** Interaction energy between mixed oil and water molecules with 3 wt% of water content.

VO Proportion	0 wt%	10 wt%	20 wt%	30 wt%	40 wt%	50 wt%
*E_int-M_* (kcal/mol)	−260.0	−234.8	−214.9	−184.7	−144.6	−124.2
*E_int-V_* (kcal/mol)	--	−80.3	−141.0	−221.0	−317.2	−343.5
*E_int-T_* (kcal/mol)	−260.0	−315.1	−355.9	−405.7	−461.8	−467.8

**Table 14:** The average interaction energy of a single H_2_O molecule and a single VO molecule.

VO Proportion	0 wt%	10 wt%	20 wt%	30 wt%	40 wt%	50 wt%
*E_per-int-V-1wt%_* (kcal/mol)	0	−0.0004	−0.0014	−0.0555	−0.1111	−0.3993
*E_per-int-V-2wt%_* (kcal/mol)	0	−0.0006	−0.0020	−0.0582	−0.2105	−0.5124
*E_per-int-V-3wt%_* (kcal/mol)	0	−0.0009	−0.0027	−0.0640	−0. 3487	−0.6579

**Table 15:** The FFV of H_2_O molecules in each mixed oil model with 1 wt% water content.

VO Proportion	0 wt%	10 wt%	20 wt%	30 wt%	40 wt%	50 wt%
Free Volume	24924.65	22889.38	21498.63	19727.60	18442.04	17084.73
Occupied Volume	176865.57	177996.34	180341.30	181149.66	181729.67	183784.16
FFV	0.124	0.114	0.107	0.098	0.092	0.085

**Table 16:** The FFV of H_2_O molecules in each mixed oil model with 2 wt% water content.

VO Proportion	0 wt%	10 wt%	20 wt%	30 wt%	40 wt%	50 wt%
Free Volume	24806.84	23229.05	21717.70	19543.77	18569.38	17006.71
Occupied Volume	178341.31	179655.43	182086.55	183997.39	184537.01	186561.44
FFV	0.122	0.114	0.106	0.096	0.091	0.084

**Table 17:** The FFV of H_2_O molecules in each mixed oil model with 3 wt% water content.

VO Proportion	0 wt%	10 wt%	20 wt%	30 wt%	40 wt%	50 wt%
Free Volume	24930.91	23396.14	21674.92	19649.61	18654.19	16706.58
Occupied Volume	181070.74	183157.74	184156.87	185236.15	187626.82	188827.81
FFV	0.121	0.113	0.105	0.095	0.090	0.081

## Data Availability

Not applicable.

## Supplementary Materials

The following supporting information can be downloaded at: https://www.mdpi.com/article/10.3390/molecules28031067/s1. Figure S1: The energy fluctuations as a function of time in the processes of (a) Annealing, (b) Structural optimization and relaxations in NPT ensemble, (c) Structural optimization and relaxations in NVT ensemble, and (d) Molecular dynamics simulation in NVT; Figure S2: Simulation Time-Dependent Test for MSDs of Water Molecules in Mixed-Oil Models; Figure S3–S5: Free volume distribution of H_2_O molecules in each mixed oil model with different water contents. (a) 0 wt% VO, (b) 10 wt% VO, (c) 20 wt% VO, (d) 30 wt% VO, (e) 40 wt% VO, (f) 50 wt% VO; Figure S6–S8: Centroid Trajectories of H_2_O molecules in each mixed oil model with different water contents: (a) 0 wt% VO, (b) 10 wt% VO, (c) 20 wt% VO, (d) 30 wt% VO, (e) 40 wt% VO, (f) 50 wt% VO.
