# Supplementary material for: Molecular Dynamics Study on the Diffusion Behavior of Water Molecules and the Dielectric Constant of Vegetable/Mineral Oil Blends
Source: Molecules. 2023 Jan 20;28(3):1067. doi: 10.3390/molecules28031067 (PMC9921694; doi:10.3390/molecules28031067)
Supplement: Supplementary file 1 [file molecules-28-01067-s001.zip › molecules-2132782-supplementary.pdf]

Molecular Dynamics Study on the Diffusion Behavior of Water Molecules and the Dielectric Constant of Vegetable/Mineral Oil Blends

Manqing Zhao [1], Bo Zhang [1,*], Jianfei Li [1], Qiankai Zhang [1] and Huaqiang Li [2,*]

[1] School of Electronics and Information, Xi’an Polytechnic University, Xi’an 710048, China;

[2] State Key Laboratory of Electrical Insulation and Power Equipment, Xi’an Jiaotong University, Xi’an 710049, China

* Correspondence: zhangbo@xpu.edu.cn (B.Z.); lhqxjtu@xjtu.edu.cn (H.L.)

1.COMPASS Force filed Definition and Parameterization

We used COMPASS (Condensed-phase Optimized Molecular Potentials for Atomistic Simulation Studies) [S1-S5] force field in our simulation reported in this paper, which is a member of the consistent family of force fields (CFF91, PCFF, CFF and COMPASS), and are closely related second-generation force fields. They were parameterized against a wide range of experimental observables for organic compounds containing H, C, N, O, S, P, halogen atoms and ions, alkali metal cations, and several biochemically important divalent metal cations. However, it is a commercial force field with its full parameters encrypted in Material Studio. Part of the parametrizations in COMPASS force filed were accessible for Alkane, Benzene Compounds and Nitrate Esters in reference [S1-S3] as listed below. The potential forms, however, were available for COMPASS in software introduction and some publications[S1-S5].

Citation: Zhao, M.; Zhang, B.; Li, J.; Zhang, Q.; Li, H. Molecular Dynamics Study on the Diffusion Behavior of Water Molecules and the Dielectric Constant of Vegetable/Mineral Oil Blends. *Molecules* **2023**, *28*, 1067. https://doi.org/10.3390/molecules28031067

Academic Editor: Dmitri B. Kireev

Received: 15 December 2022
Revised: 8 January 2023
Accepted: 18 January 2023

Copyright: © 2023 by the authors. Submitted for possible open access publication under the terms and conditions of the Creative Commons Attribution (CC BY) license (https://creativecommons.org/licenses/by/4.0/).

ε_{ij}

The functional form of CAMPASS force filed is composed of two parts: the bonding term and the non-bonding term. The bonding term includes diagonal and non-diagonal cross-coupling terms, as shown in Eq. S1, which are bond stretching energy, bond Angle bending energy, bond torsion energy, bond Angle out of plane bending energy and their mutual coupling energy. The non-bonding terms include van der Waals energy (Eq. S2) and Coulomb energy (Eq. S3), which are used to calculate the interaction forces between two or more pairs of atoms at a distance or on different molecular chains.

$$\begin{aligned}
E_B &= E_b + E_\theta + E_\Phi + E_\chi + E_{cross} \\
&= \sum_b \left[k_2 (b - b_0)^2 + k_3 (b - b_0)^3 + k_4 (b - b_0)^4 \right] \\
&+ \sum_\theta \left[k_2 (\theta - \theta_0)^2 + k_3 (\theta - \theta_0)^3 + k_4 (\theta - \theta_0)^4 \right] \\
&+ \sum_\Phi \left[k_1 (1 - \cos\Phi) + k_2 (1 - \cos 2\Phi) + k_3 (1 - \cos 3\Phi) \right] \\
&+ \sum_\chi k_2 \chi^2 + \sum_{bb'} k (b - b_0)\left(b - b'_0\right) + \sum_{b\theta} k (b - b_0)(\theta - \theta_0) \\
&+ \sum_{b'\Phi} (b - b_0)\left[k_1 \cos\Phi + k_2 \cos 2\Phi + k_3 \cos 3\Phi\right] \\
&+ \sum_{b'\Phi} \left(b' - b'_0\right)\left[k_1 \cos\Phi + k_2 \cos 2\Phi + k_3 \cos 3\Phi\right] \\
&+ \sum_{\theta\Phi} (\theta + \theta)\left[k \cos\Phi + k \cos 2\Phi + k \cos 3\Phi\right] \\
&+ \sum_{\theta'\theta} k \left(\theta' - \theta'_0\right)(\theta - \theta_0) + \sum_{\theta'\theta\Phi} k (\theta - \theta_0)\left(\theta' - \theta'_0\right)\cos\Phi
\end{aligned} \tag{S1}$$

$$Eij = \sum_{ij} \varepsilon_{ij} \left[2\left(\frac{r_{ij}^0}{r_{ij}}\right)^9 - 3\left(\frac{r_{ij}^0}{r_{ij}}\right)^6 \right] \tag{S2}$$

$$E_{elec} = \sum_{ij} \frac{q_i q_j}{r_{ij}} \tag{S3}$$

The van der Waals interaction uses the 9-6 Lennard-Jones potential, (also known as 6-9 potential) for the parameters between pairs of atoms of different species r_{ij} , ε_{ij} can be based on the same atomic pair parameters ε, r^0 using the sixth power average method can to calculate.

$$r_{ij}^{\ 0} = \left(\frac{\left(r_i^0\right)^6 - \left(r_i^0\right)^6}{2} \right)^{1/6} \quad \varepsilon_{ij} = 2\sqrt{\varepsilon_i \cdot \varepsilon_j} \left(\frac{\left(r_i^0\right)^3 \cdot \left(r_j^{\ 0}\right)^3}{\left(r_i^0\right)^6 \cdot \left(r_j^{\ 0}\right)^6} \right) \tag{S4}$$

Electrostatic interactions are described in terms of atomic residual charges, in which the bond increment δ_{ij} is used to represent the residual charge of atom *j* to atom *i* (*i* is the charge acceptor, *j* is the charge donor). The charge bond increment delta δ_{ij} is obtained by ab initio calculation of electrostatic potential energy. As shown in Eq. S5, the residual charge of atom i is the sum of bond increments delta δ_{ij} of all the charges bonding with it.

$$q_i = \sum \delta_{ij} \tag{S5}$$

COMPASS meter uses a simple ionic mode, which includes the electrostatic term (Eq. S2) and van der Waals term (Eq. S3). In this model, each atom acts as a non-bonded particle, i.e., there is no special topological connection mode and special distance between ions. The structure of the particles depends on the strong electrostatic attraction and van der Waals repulsion between different ions. In this model, atomic charge is not determined by means of charge difference at bond end.

2. Energy Fluctuations During Molecular Dynamics Simulation

The energy change curves of each step in the simulation process are drawn, as shown in Figure S1. (a) is the energy change curve in the annealing optimization process, (b) is the energy change curve in the dynamic optimization process of the ensemble with NVT, (c) is the energy change curve in the dynamic optimization process of the ensemble with NPT, and (d) is the energy change curve in the dynamic simulation process.

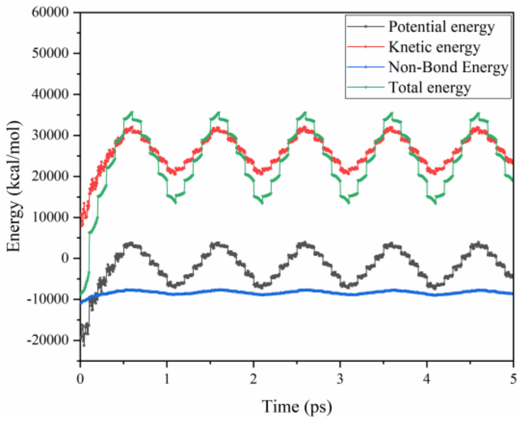

(a) Annealing

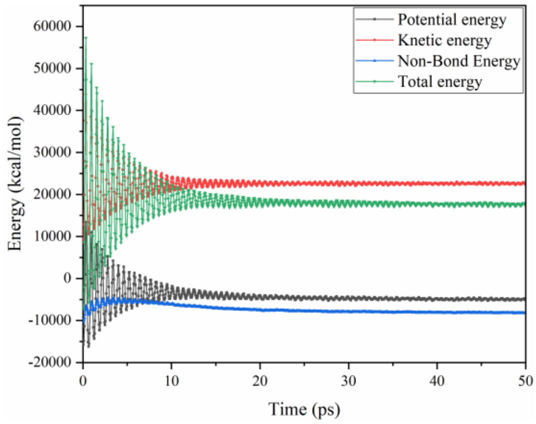

(b) Optimizations and relaxations in NVT

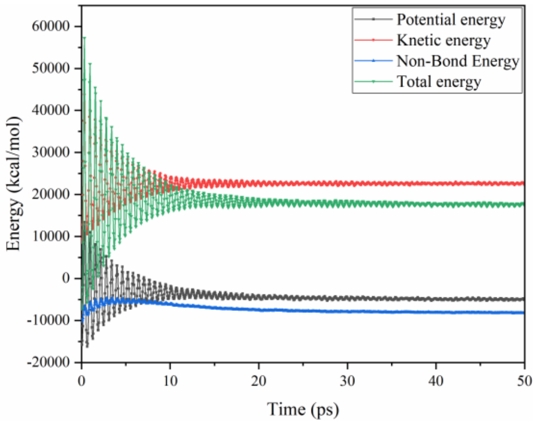

(c) Optimizations and relaxations in NPT

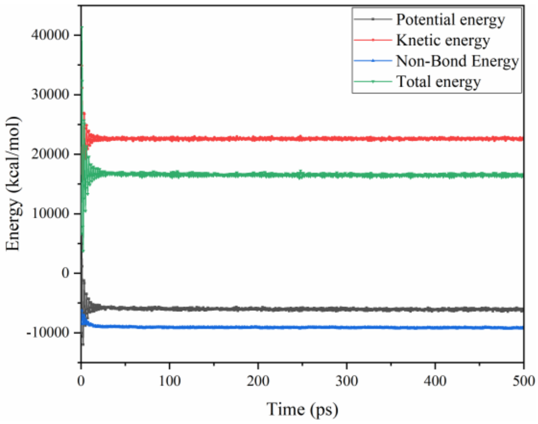

(d) Molecular dynamics simulation in NVT

Figure S1. The energy fluctuations as a function of time in the processes of (a) Annealing, (b) Structural optimization and relaxations in NPT ensemble, (c) Structural optimization and relaxations in NVT ensemble, and (d) Molecular dynamics simulation in NVT.

The initial structures were subjected to five cycles of annealing between 343 K and 500 K in order to overcome the restrictions introduced by the dihedral potential barrier of the molecular chains. Therefore, the energy fluctuations were in the same variation pattern as its annealing temperature variations. The energy fluctuations as a function of time in the processes of structural optimization and relaxations as shown in Figure S1 (b) and (c), as well as in the process of molecular dynamics simulation in NVT ensemble all reached a plateau after a short period of time, indicating the energy was conserved during the above simulation processes and the system were gradually reached its equilibrium.

3. Simulation Time-Dependent Test for Mean Square Displacement (MSD) of Water Molecules in Mixed-Oil Models

In this section, we tested the influence of the duration (500 ps and 1500 ps) of molecular dynamics simulation on the result of MSD, and analyzed the energy curve during the simulation.

It could be seen from Figure S2 (a) and (b) that regardless of simulation times, the MSD curves both exhibit peculiar pattern at the end of the simulations in both trajectories calculated by MS. This irregular behavior of MSD happened at the end of the simulation, approximated within the last 100 ps in the simulation. The initial 400 ps of MSD curves calculated by 0.5 ns and 1.5 ns of the same trajectory coincide with each other in both cases. Moreover, the energy fluctuations all reached a plateau with in a short amount of time regardless of different simulation time and different simulation trajectories.

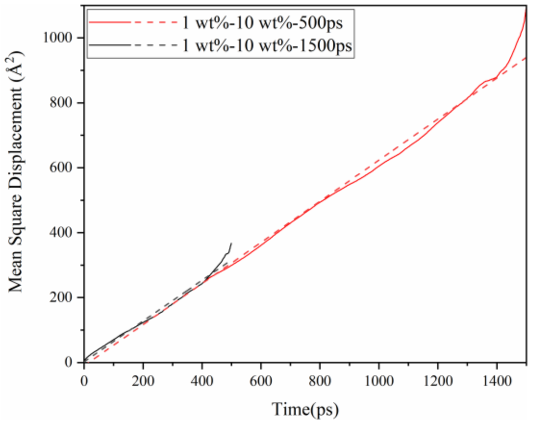

(**a**) The MSD curves of water molecules with simulation time of 0.5 ns and 1.5 ns at 1 wt% water content

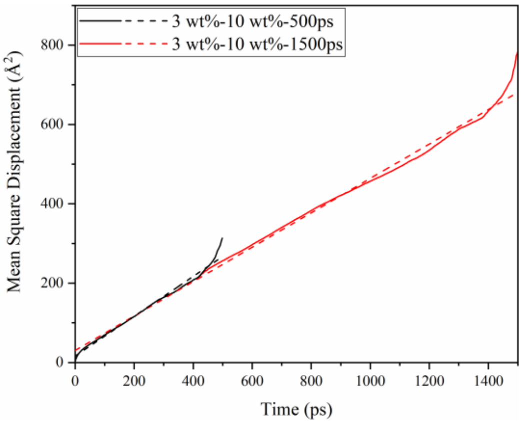

(**b**) The MSD curves of water molecules with simulation time of 0.5 ns and 1.5 ns at 3 wt% water content

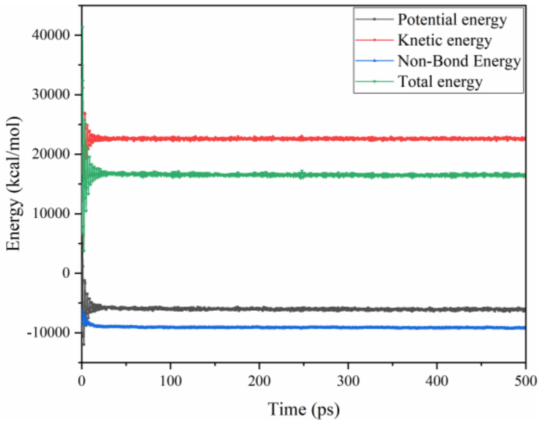

(**c**) The energy fluctuations as a function of time with 0.5 ns simulation time at 1 wt% water content in NVT

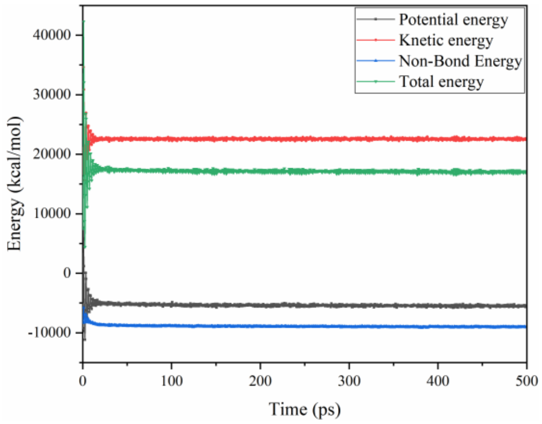

(**d**) The energy fluctuations as a function of time with 0.5 ns simulation time at 1 wt% water content in NVT

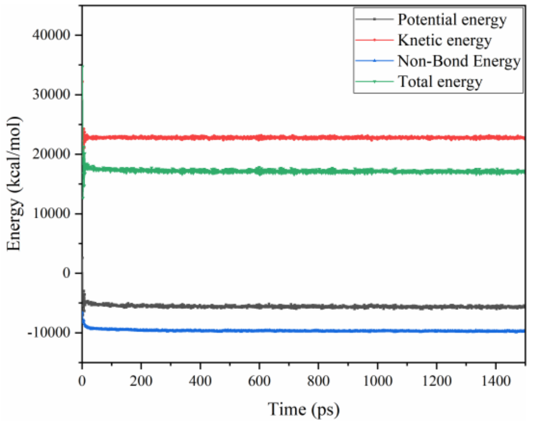

(**e**) The energy fluctuations as a function of time with 1.5 ns simulation time at 1 wt% water content in NVT

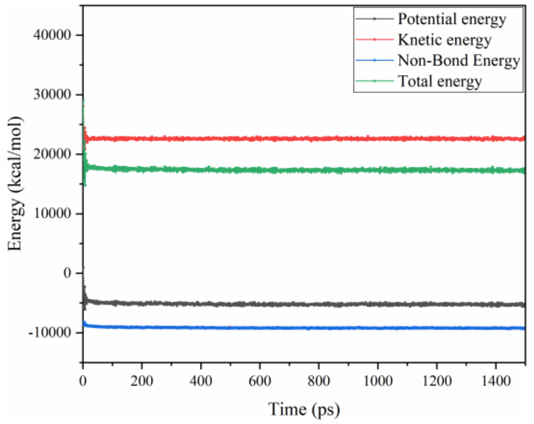

(**f**) The energy fluctuations as a function of time with 1.5 ns simulation time at 3 wt% water content in NVT

Figure S2. Simulation Time-Dependent Test for MSDs of Water Molecules in Mixed-Oil Models.

Our simulation time-dependent test for MSDs of water molecules in mixed oil models confirmed that the peculiar sharp increase behavior at the end of the simulation were caused by MS software MSD calculation codes. In order to eliminate the error in MSDs introduced by the last 100 ps calculation, we performed a successive MD simulation in of 100 ps in conjunction with our previous 500 ps calculations in 18 different independent trajectories and recalculated the MSD curves using 600 ps trajectories. And we delete the last 100 ps part of MSD, leaving the former 500 ps calculation results for the effective MSD results for calculating the diffusion coefficients in different cases, in order to reduce the calculation errors

introduced by the code.

4. Fractional Free Volume (FFV)

The free volume distribution of water molecules under 1 wt%, 2 wt% and3 wt% water contentin the mixed oil system is shown in Figure S4, S5 and S6. The free volume can reflect the space for water molecules to diffuse in the mixed oil system to some extent, further reflecting the diffusion capacity of water molecules.

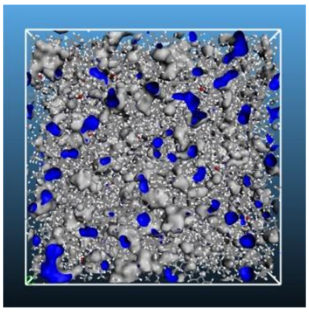
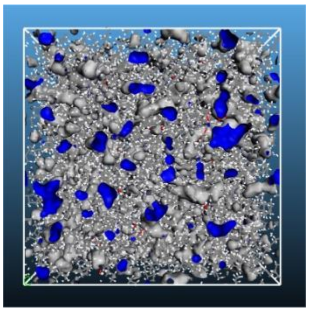
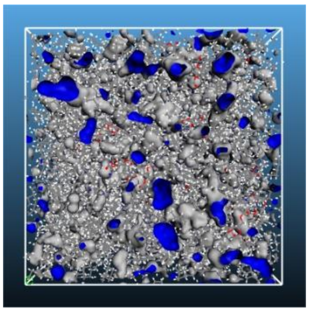

(**a**) 0 wt% (**b**) 10 wt% (**c**) 20 wt %

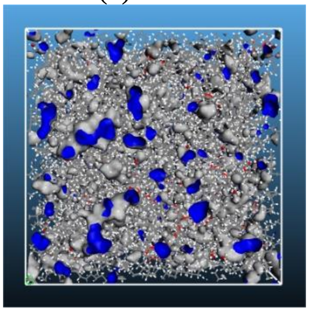
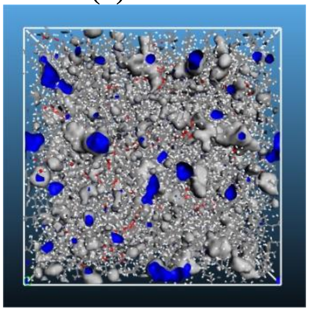
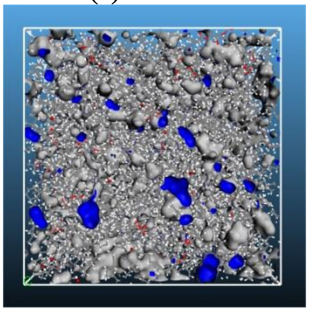

(**d**) 30 wt% (**e**) 40 wt% (**f**) 50 wt%

Figure S3. Free volume distribution of H_2O molecules in each mixed oil model with 1 wt% water content. (**a**) 0 wt% VO; (**b**) 10 wt% VO; (**c**) 20 wt% VO; (**d**) 30 wt% VO; (**e**) 40 wt% VO; (**f**) 50 wt% VO.

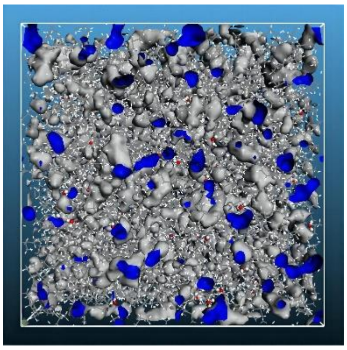
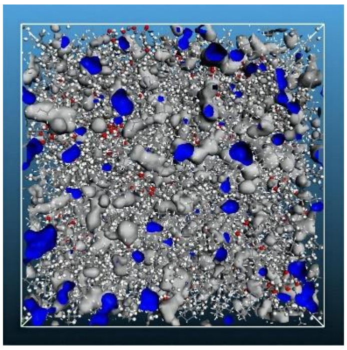
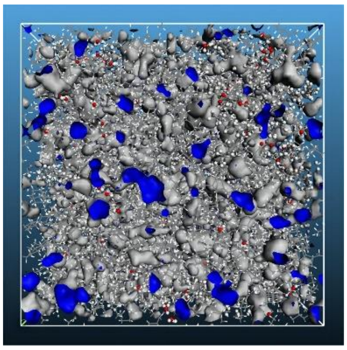

(**a**) 0 wt% (**b**) 10 wt% (**c**) 20 wt%

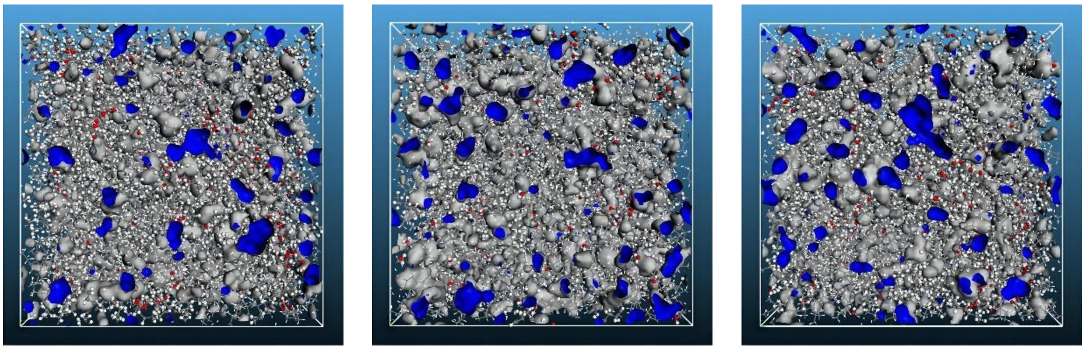

(**d**) 30 wt% (**e**) 40 wt% (**f**) 50 wt%

Figure S4. Free volume distribution of H_2O molecules in each mixed oil model with 2 wt% water content. (**a**) 0 wt% VO; (**b**) 10 wt% VO; (**c**) 20 wt% VO; (**d**) 30 wt% VO; (**e**) 40 wt% VO; (**f**) 50 wt% VO.

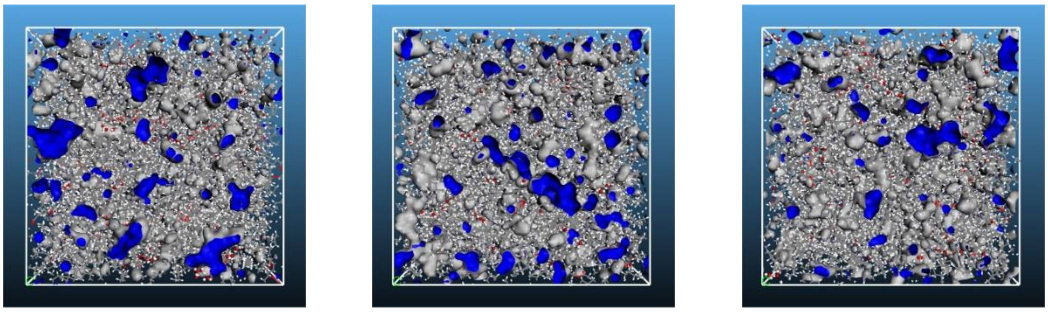

(**a**) 0 wt% (**b**) 10 wt% (**c**) 20 wt%

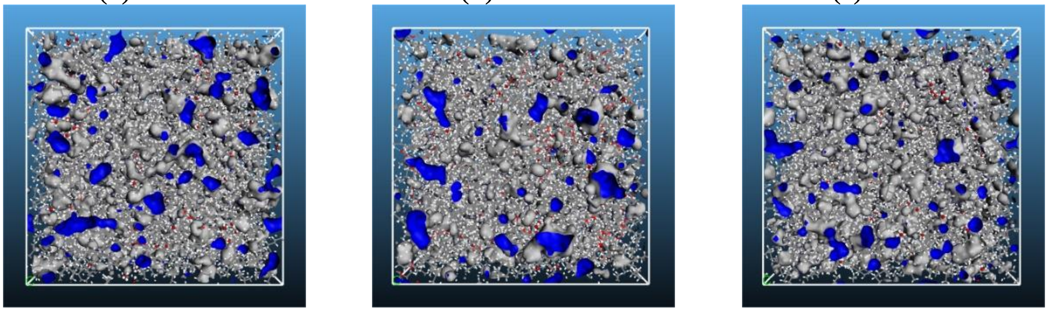

(**d**) 30 wt% (**e**) 40 wt% (**f**) 50 wt%

Figure S5. Free volume distribution of H_2O molecules in each mixed oil model with 3 wt% water content. (**a**) 0 wt% VO; (**b**) 10 wt% VO; (**c**) 20 wt% VO; (**d**) 30 wt% VO; (**e**) 40 wt% VO; (**f**) 50 wt% VO.

5. Centroid Trajectories of Water Molecules

The centroid locus of water molecules with 1 wt%, 2 wt% and 3 wt% moisture content is shown in Figure S7, S8, and S9. The centroid locus can visually reflect the movement of water molecules in the mixed oil system, and thus reflect the diffusion capacity of water molecules.

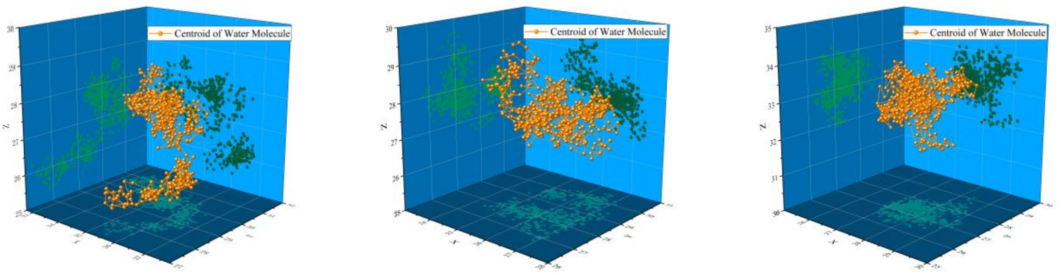

(**a**) 0 wt% (**b**) 10 wt% (**c**) 20 wt%

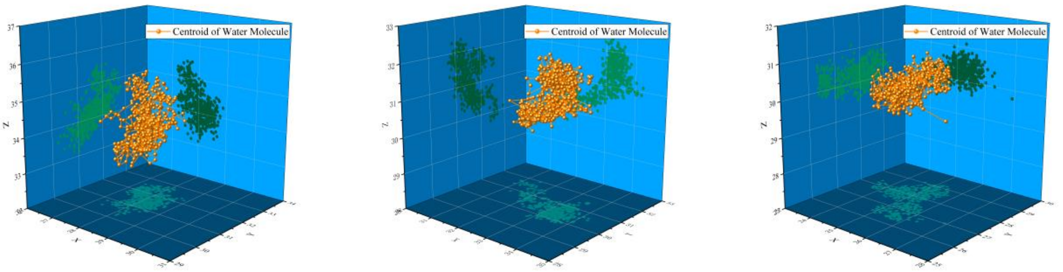

(**d**) 30 wt% (**e**) 40 wt% (**f**) 50 wt%

Figure S6. Centroid Trajectories of H_2O molecules in each mixed oil model with 1 wt% water content: (**a**) 0 wt% VO; (**b**) 10 wt% VO; (**c**) 20 wt% VO; (**d**) 30 wt% VO; (**e**) 40 wt% VO; (**f**) 50 wt% VO.

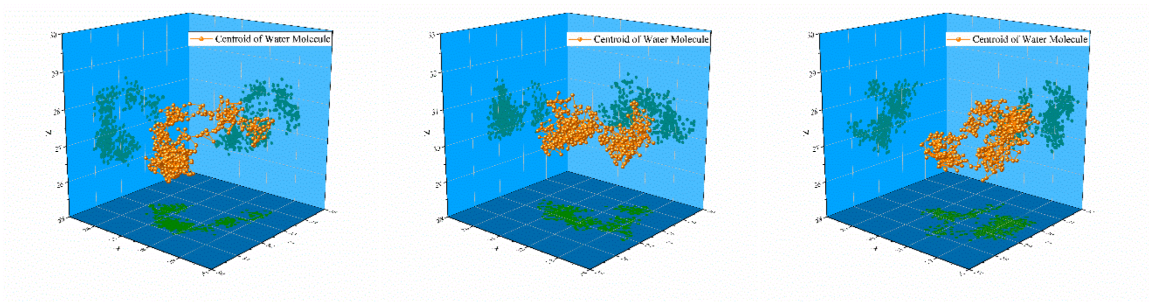

(**a**) 0 wt% (**b**) 10 wt% (**c**) 20 wt%

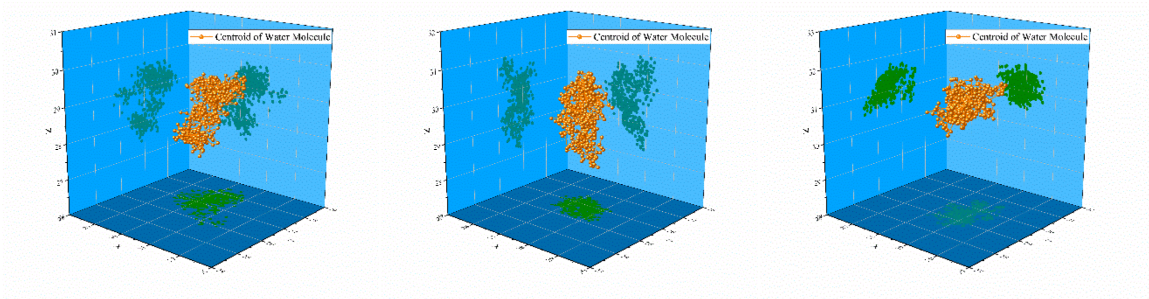

(**d**) 30 wt% (**e**) 40 wt% (**f**) 50 wt%

Figure S7. Centroid Trajectories of H_2O molecules in each mixed oil model with 2 wt% water content: (**a**) 0 wt% VO; (**b**) 10 wt% VO; (**c**) 20 wt% VO; (**d**) 30 wt% VO; (**e**) 40 wt% VO; (**f**) 50 wt% VO.

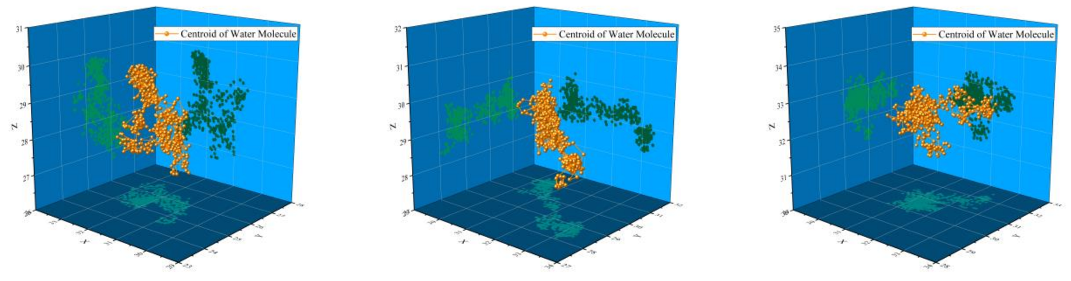

(**a**) 0 wt% (**b**) 10 wt% (**c**) 20 wt%

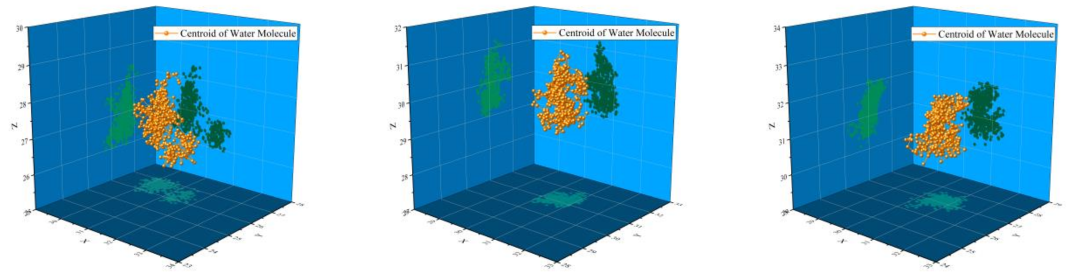

(**d**) 30 wt% (**e**) 40 wt% (**f**) 50 wt%

Figure S8. Centroid Trajectories of H_2O molecules in each mixed oil model with 3 wt% water content: (**a**) 0 wt% VO; (**b**) 10 wt% VO; (**c**) 20 wt% VO; (**d**) 30 wt% VO; (**e**) 40 wt% VO; (**f**) 50 wt% VO.

References:

[S1] Sun H. COMPASS: An ab Initio Force-Field Optimized for Condensed-Phase Applications - Overview with Details on Alkane and Benzene Compounds. Journal of Physical Chemistry B 1998, 102, 7338–7364.

[S2] Sun H., Ren P., Fried J. The COMPASS force field: parameterization and validation for phosphazenes. Computational and Theoretical Polymer Science 1998, 8, 229-246.

[S3] Bunte S., Sun H. Molecular Modeling of Energetic Materials: The Parameterization and Validation of Nitrate Esters in the COMPASS Force Field. Journal of Physical Chemistry B 2000, 104, 2477-2489.

[S4] Yang J., Ren Y., Tian A., Sun H. COMPASS Force Field for 14 Inorganic Molecules, He, Ne, Ar, Kr, Xe, H_2, O_2, N_2, NO, CO, CO_2, NO_2, CS_2, and SO_2, in Liquid Phases. Journal of Physical Chemistry B 2000, 104, 4951-4957.

[S5] McQuaid M., Sun H., Rigby D. Development and validation of COMPASS force field

parameters for molecules with aliphatic azide chains. Journal of Computational Chemistry 2004, 25, 61-71.
